# Approaching treatment of male infertility: the APHRODITE criteria

**DOI:** 10.1590/S1677-5538.IBJU.2024.9908

**Published:** 2024-03-10

**Authors:** Sandro C. Esteves, Peter Humaidan

**Affiliations:** 1 ANDROFERT - Clínica de Andrologia e Reprodução Humana Campinas SP Brasil ANDROFERT - Clínica de Andrologia e Reprodução Humana, Campinas, SP, Brasil; 2 Disciplina de Urologia da Universidade Estadual de Campinas Departamento de Cirurgia Campinas SP Brasil Departamento de Cirurgia - Disciplina de Urologia da Universidade Estadual de Campinas (UNICAMP), Campinas, SP, Brasil; 3 Aarhus University Faculty of Health Department of Clinical Medicine Denmark Faculty of Health, Department of Clinical Medicine, Aarhus University, Aarhus, Denmark; 4 Skive Regional Hospital Fertility Unit Skive Denmark Skive Regional Hospital, Fertility Unit, Skive, Denmark

## INTRODUCTION

We have the privilege to introduce a groundbreaking advancement in the field of male infertility to the readers of IBJU: the APHRODITE criteria (1), short for "Addressing male Patients with Hypogonadism and/or infeRtility Owing to altereD, Idiopathic TEsticular function." Named after the Greek goddess of fertility and procreation, this novel classification system can represent a significant leap forward in our understanding and treatment of male infertility.

### The Need for Improved Classification

Male infertility is a complex clinical condition, and thus far, the clinical management of men with reduced fertility and impaired spermatogenesis has been fraught with difficulties and limited advancement (2). This has led to frustration among both clinicians and patients, perpetuating the belief that intracytoplasmic sperm injection (ICSI) is the only solution to provide the couple with a baby without the need to explore the nature or cause of the underlying male infertility (3, 4). The APHRODITE criteria aim to address this gap by providing a structured approach to characterize male infertility in men seeking paternity, particularly those who may benefit from hormonal treatment (1). Importantly, these criteria are not designed for men with established infertility diagnoses, such as varicocele, infection, or obstruction, who would not benefit from hormonal treatment (3).

### The Role of Hormones in Spermatogenesis

Understanding the APHRODITE criteria begins with a brief review of human spermatogenesis, a complex process that takes approximately 75 days and is primarily controlled by follicle-stimulating hormone (FSH) and luteinizing hormone (LH)-driven testosterone (5). These hormones are crucial in regulating spermatogenesis, making them central to our approach.

### LH and Testosterone

The hypothalamus secretes GnRH, which triggers the pituitary gland to secrete FSH and LH.

In the testicle, key cells for the action of these gonadotropins are the Sertoli and Leydig cells. LH is vital for stimulating testosterone production in Leydig cells, which, in turn, binds to androgen receptors on Sertoli cells, regulating gene transcription and supporting spermatogenesis (5). Testosterone primarily supports the transformation of round spermatids into mature sperm during the late stages of spermatogenesis. Additionally, testosterone aids in transitioning type A to type B spermatogonia and upregulates androgen receptor expression, which improves Sertoli cell function (5).

### The Vital Role of FSH

FSH works in synergy with LH and testosterone, acting on Sertoli cells to provide essential metabolic and structural support for spermatogenesis (5). FSH also controls the proliferation, growth, and maturation of Sertoli cells, and it triggers the release of androgen-binding protein. While not indispensable for the completion of spermatogenesis in humans, FSH deficiency markedly reduces sperm quantity (5, 6).

### The Underlying Causes of Hypogonadism

Patients with reduced fertility and impaired spermatogenesis may face inadequate testicular stimulation due to deficits in FSH and/or LH production or action (5). Hypogonadism, characterized primarily by insufficient testosterone production, has various causes, including testicular pathologies, systemic diseases, infections, congenital abnormalities, aging, and poor lifestyle (7). In some instances, the underlying cause remains elusive, and hypogonadism is labeled idiopathic.

### The Birth of the APHRODITE Criteria

The APHRODITE criteria emerged from the collaborative efforts of male infertility experts, including andrologists, reproductive urologists, and IVF specialists (1). Inspired by the POSEIDON concept, a stratification system developed for infertile women (8-10), these experts meticulously developed the APHRODITE criteria via an interactive consensus process, relying on clinical patient descriptions and routine laboratory tests, such as semen analysis and hormonal assessment, particularly FSH and testosterone levels (see Table-1) (1). For semen analysis, the WHO framework and reference ranges were applied (11). The FSH levels are grouped as low, normal, or high based on typical reference ranges. For testosterone, 350 ng/dL was the proposed threshold, which is endorsed by most guidelines, and below which the patient is classified as hypogonadal (1).

**Table 1:** Laboratory tests and results interpretation in the context of the APHRODITE criteria.

Semen analysis parameters	Normal	Percentages of motile and morphologically normal spermatozoa and concentration of spermatozoa in the ejaculate that is equal to or above the 5^th^ percentile of the data from the 2021 WHO semen analysis manual reference limits (WHO 2021)
	Lowered	Reduced percentages of motile and morphologically normal spermatozoa and concentration of spermatozoa in the ejaculate that is lower than the 5^th^ percentile of the data from the 2021 WHO semen analysis manual reference limits (WHO 2021)
	Azoospermia	Absence of spermatozoa in the ejaculate*
FSH level	Normal	FSH within the normal range of the assessing laboratory
	Reduced	FSH below the normal range of the assessing laboratory
	Elevated	FSH above the upper limit of the normal range of the assessing laboratory
Testosterone level	Normal	Testosterone above the lower limit of the normal range of the assessing laboratory
	Reduced	Testosterone below the lower limit of the normal range of the assessing laboratory

* After examination of the centrifuged pellet.

FSH: follicle-stimulating hormone; WHO: World Health Organization

Adapted with permission from Esteves et al. Reprod Biomed Online. 2023 Oct 29;48(4):103647. (Reference 1)

### Stratifying Male Infertility

**Figure 1:**
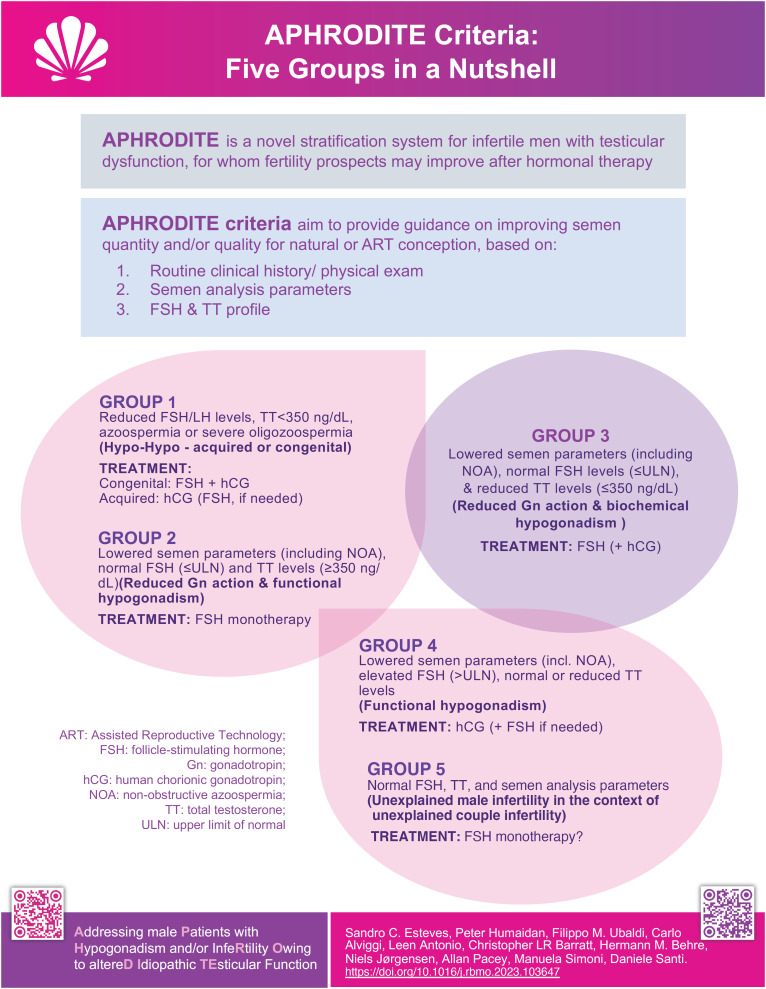
Overview of the APHRODITE Criteria and the Five Male Infertility Groups

The APHRODITE criteria categorize male infertility patients into five distinct groups, each with its characteristics and suggested therapeutic management (Figure-1; Table-2).

**Table 2:** Characteristics of the five APHRODITE groups.

Classification	Definition	Prevalence	Suggested gonadotropin regimen	Endpoints†
**Group 1 Hypogonadotropic Hypogonadism (acquired and congenital)**	Gonadal failure associated with reduced gametogenesis and reduced gonadal steroid production due to reduced gonadotropin production or action; FSH levels below reference ranges and reduced LH levels, reduced T levels (e.g., <350 ng/dL)) and lowered semen analysis parameters (e.g., OAT or azoospermia)	∼1.9% of azoospermia cases and 1.6% of male infertility cases overall	hCG (±) FSH ^1^	Semen parameters/sperm retrieval rates, T levels, QoL, pregnancy rates
**Group 2 Lowered semen analysis parameters, normal serum FSH and normal serum total testosterone**	Functional hypogonadism with reduced gonadotropin action; Lowered semen analysis parameters, including NOA; FSH levels within the reference range and normal T levels (e.g., >=350 ng/dL)	Idiopathic male infertility: Up to 44% of male infertility cases	FSH alone	Semen parameters /sperm retrieval rates, SDF rates, QoL, pregnancy rates
**Group 3 Lowered semen analysis parameters, normal FSH and reduced total testosterone levels**	Functional hypogonadism with reduced gonadotropin action and reduced T production; Lowered semen analysis parameters, including NOA; FSH levels within the reference range and reduced T (e.g. <350 ng/dL)	∼20% of the total idiopathic male infertility population treated with hormonal therapy)	FSH ^2^ * (± hCG)	Semen parameters/sperm retrieval rates, T levels, SDF rates, QoL, pregnancy rates
**Group 4 Lowered semen analysis parameters, elevated FSH levels, and normal or reduced total testosterone levels**	Functional hypergonadotropic hypogonadism; Lowered semen analysis parameters, mainly NOA; FSH levels above the upper limit of the reference range and normal or reduced T levels (excluding genetic causes)	Up to 10%	hCG (± FSH ^2^ *)	Semen parameters/sperm retrieval rates, T levels, SDF rates, QoL, pregnancy rates
**Group 5 Unexplained male infertility in the context of unexplained couple infertility**	FSH levels within the reference range, T levels within the normal range, and normal semen analysis parameters	15% of couples presenting with unexplained infertility, and unexplained male infertility from 6–27%	FSH alone**	SDF rates, pregnancy rates

FSH, follicle-stimulating hormone. hCG, human chorionic gonadotropin. LH, luteinizing hormone. T, total testosterone

NOA, non-obstructive azoospermia. OAT, oligoasthenoteratospermia. QoL, quality of life. SDF, Sperm DNA fragmentation.

* FSH treatment can improve DNA fragmentation and sperm quality

† Semen parameters are the primary outcome of hormonal treatment.

1 Regimen can be tailored according to the congenital or acquired forms of HH

** The suggestion for FSH alone is based on empirical evidence and the authors' opinion and will need to be updated as more data becomes available.

2 Groups 2 and 3 (Italy only) would meet the label for FSH treatment. Groups 4 and 5 would be off-label treatment.

Adapted from: Esteves et al. Reprod Biomed Online. 2023 Oct 29;48(4):103647, with permission (Reference 1).

### Aphrodite Group 1: Hypogonadotropic Hypogonadism

This group comprises patients with congenital or acquired hypogonadotropic hypogonadism (12). They present with a hormonal disorder caused by deficient gonadotropin production, which prevents their testicles from producing sperm. Typically, these patients have low FSH, LH, and testosterone levels, usually combined with azoospermia or, less frequently, severe oligozoospermia. Gonadotropin therapy with hCG and FSH can restore spermatogenesis in up to 90 percent of these patients, offering hope for fatherhood (5).

### Aphrodite Group 2: Idiopathic Male Infertility

This group predominantly encompasses patients with idiopathic oligozoospermia (≤ 16 million spermatozoa per ml) and select cases of nonobstructive azoospermia (5, 13). These individuals exhibit abnormal semen analysis, a normal physical examination, and normal laboratory results, suggestive of functional hypogonadism. FSH therapy has shown promise in improving semen parameters and pregnancy rates (5), and it might also work in some patients with non-obstructive azoospermia (5).

### Aphrodite Group 3: Biochemical Hypogonadism

This group shares similarities with Group 2 but differs by exhibiting reduced testosterone levels, indicating biochemical hypogonadism (1, 7). Combining hCG with FSH may be beneficial in these patients as hCG boosts intratesticular testosterone production. Some patients with nonobstructive azoospermia fitting this group have experienced improvements in sperm retrieval rates after hormonal treatment (14).

### Aphrodite Group 4: Hypergonadotropic Hypogonadism

Group 4 primarily encompasses patients with nonobstructive azoospermia characterized by high FSH and low testosterone levels, indicating hypergonadotropic hypogonadism (7). These individuals have low testicular reserve, making them a challenge to treat (15). Nonetheless, a few observational studies have shown that hormonal treatment improves sperm retrieval rates in some cases (5, 16).

### Aphrodite Group 5: Unexplained Male Infertility

The final group consists of patients with unexplained infertility, showing no history of diseases affecting fertility, and also normal semen analysis parameters, physical examination, and laboratory findings. It has been postulated that FSH stimulation might benefit these patients by enhancing spermatogenesis; the hypothesis being that spermatogenesis does not run at its maximum capacity, and additional FSH stimulation could potentially boost spermatogenesis (17). It is noteworthy that in couples attempting natural conception, higher sperm concentrations and total sperm counts are associated with a shorter time to pregnancy (18, 19). However, research is warranted to validate the hypothesis of testicular hyperstimulation.

### Challenges and Opportunities

While the existing evidence supports the efficacy of gonadotropin therapy in Aphrodite Groups 1, 2, and 3, the available data remains limited. Larger, well-designed studies are necessary to confirm the clinical utility in these groups and to further explore the potential in Groups 4 and 5. Besides gonadotropins, other therapeutic modalities like selective estrogen-receptor modulators and aromatase inhibitors could be explored to modulate reproductive hormones. The APHRODITE criteria have the potential to facilitate communication among clinicians, researchers, and patients and, most importantly, to enhance reproductive outcomes through hormonal therapy. APHRODITE criteria are also suggested to pave the way for future clinical trials of hormonal treatment in male infertility, offering hope to countless couples.

## CONCLUSIONS

In summary, the APHRODITE criteria significantly advance the stratification and management of male infertility. The criteria provide a clear and well-defined system, classifying patients and promoting communication among healthcare providers, researchers, and patients. Moreover, these criteria open doors to research into new pharmacological interventions and the discovery of novel causes of male infertility.
